# Histopathological, Immunohistochemical, Biochemical, and In Silico Molecular Docking Study of Fungal-Mediated Selenium Oxide Nanoparticles on *Biomphalaria alexandrina* (Ehrenberg, 1831) Snails

**DOI:** 10.3390/microorganisms11030811

**Published:** 2023-03-22

**Authors:** Amina M. Ibrahim, Mahassen Ghazy, Heba El-Sayed, Rehab M. Abd El-Hameed, Rehab G. Khalil, Shereen M. Korany, Abeer S. Aloufi, Olfat A. Hammam, Mostafa Y. Morad

**Affiliations:** 1 Medical Malacology Department, Theodor Bilharz Research Institute, Giza 12411, Egypt; 2 Water Pollution Research Department, National Research Centre, El Tahrir Street, Dokki, Giza 12622, Egypt; 3 Botany and Microbiology Department, Faculty of Science, Helwan University, Helwan 11795, Egypt; 4 Immunology Division, Faculty of Science, Beni-Suef University, Beni-Suef 62521, Egypt; 5 Department of Biology, College of Science, Princess Nourah bint Abdulrahman University, P.O. Box 84428, Riyadh 11671, Saudi Arabia; 6 Pathology Departments, Theodor Bilharz Research Institute, Giza 12411, Egypt; 7 Zoology and Entomology Department, Faculty of Science, Helwan University, Helwan 11795, Egypt

**Keywords:** *Biomphalaria alexandrina*, selenium oxide nanoparticles, *Penicillium chrysogenum*, *Daphnia magna*, reproductive rate, biochemical, immunohistopathology, molecular docking

## Abstract

*Daphnia magna* and freshwater snails are used as delicate bioindicators of contaminated aquatic habitats. Due to their distinctive characteristics, selenium oxide nanoparticles (SeONPs) have received interest regarding their possible implications on aquatic environments. The current study attempted to investigate the probable mechanisms of fungal-mediated selenium nanoparticles’ ecotoxicological effects on freshwater *Biomphalaria alexandrina* snails and *Daphnia magna*. SeONPs revealed a toxicological impact on *D. magna*, with a half-lethal concentration (LC_50_) of 1.62 mg/L after 24 h and 1.08 mg/L after 48 h. Survival, fecundity, and reproductive rate were decreased in *B. alexandrina* snails exposed to SeONPs. Furthermore, the aspartate aminotransferase (AST) and alanine aminotransferase (ALT) levels were markedly elevated, while albumin and total protein levels decreased. Histopathological damage in the hermaphrodite and digestive glands was detected by light, electron microscopy, and immunohistochemistry studies. The molecular docking study revealed interactions of selenium oxide with the ALT and AST. In conclusion, *B. alexandrina* snails and *D. magna* could be employed as bioindicators of selenium nanomaterial pollution in aquatic ecosystems. This study emphasizes the possible ecological effects of releasing SeONPs into aquatic habitats, which could serve as motivation for regulatory organizations to monitor and control the use and disposal of SeONPs in industry.

## 1. Introduction

Nanotechnology development in a wide range of industries, including chemical, electronics, metal industry, biomedicine, cosmetics, and household purposes, poses a potential threat to the natural environment. Nanoparticles (NPs) are frequently utilized in a variety of industrial applications and are released into the environment through wastewater from houses and different industries. They are extremely harmful to aquatic life [1].

One of those elements that has attracted interest in the industrial and biomedical sector is elemental selenium (Se), which can be found in the form of selenium nanoparticles (SeNPs). Selenium is a vital element for life and can be found in nature in four different forms: selenate (+VI, SeO_4_^2−^), selenite (+IV, SeO_3_^2−^), elemental selenium (0, Se^0^), and selenide (−II, Se^2−^) [2]. Se toxicity varies with dosage and chemical types; dangerous Se concentrations have an adverse impact on the ecosystem [3,4]. Due to their antioxidant characteristics and ability to be integrated into selenoproteins, SeNPs have been used in several therapeutic applications, including the cancer treatment and diabetes. [5,6]. They have also been shown to suppress antibiotic-resistant bacterial strains at levels as low as 1 ppm [6]. Furthermore, SeNPs can be employed in glass production and in screening mammography detectors, and have been applied in a number of industries, including inverters and solar cells [7,8]. SeNPs are naturally occurring in the ecosystem, and living organisms, such as plants, fungi, or soil bacteria, can produce biogenic SeNPs by reducing selenite or selenate ions to these nanoparticles. Synthesized SeNPs are then probable to exist and have been distributed to numerous aquatic ecosystems. Although it has widely used applications, selenium nanoparticles’ potential for bioaccumulation makes it an effective harmful element for natural ecosystems and public health since it releases ROS and causes toxicity [9]. While selenium is naturally found in the crust of the earth, around 40% of selenium emissions into the atmosphere and aquatic habitats are caused by various industrial processes, particularly those associated with mining [8]. However, little is known about its toxicity and how functionalization affects aquatic invertebrates [10].

The second-most abundant animal group, the Mollusca phylum, is often regarded as a great bioindicator of ecosystem health since they are sensitive, accessible, and common. They are also commonly employed in environmental studies. The OECD has designed and approved *Lymnaea stagnalis*, a freshwater pulmonate snail, as a viable candidate species for assessing the effects of chemical pollutants on reproduction in Testing Series, No. 121 [11,12]. Snails (Phylum Mollusca, Class Gastropoda) have gained great attention due to their role in ecosystems and disease transmission [13]. *Biomphalaria alexandrina* (Ehrenberg, 1831) is a common freshwater snail in Egypt [14]. It is a host for the trematode parasite *Schistosoma manosni*, which causes human schistosomiasis [15]. It is a well-known model for investigating the toxicity and toxicokinetics of inorganic materials in aquatic ecosystems [16]. These species serve as an important biomonitor for pollution caused by heavy metals [17].

Freshwater zooplankton belonging to the Cladocera order, *Daphnia magna* (Straus, 1820), is found in the aquatic environments of Egypt [18]. As a non-target organism, it is a highly useful model to evaluate the chronic and acute toxicity in freshwater ecosystems using *B. alexandrina* habitat [19,20]. In addition, it is a highly sensitive animal to many minerals and organic contaminants [21]. Its laboratory culture is simple, and it has a parthenogenetic reproduction mode and a high fertility rate [19].

The enzymes known as aspartate aminotransferase (AST) and alanine aminotransferase (ALT) are mostly located in the liver, red blood cells, heart, kidneys, and pancreatic cells [22]. The enzyme activity in the liver is frequently utilized as indicators for hazardous substances. Tissue damage is directly correlated with blood levels of the AST and ALT enzymes. The tissue damages increase or decrease depending on the concentration of the enzymes present [23]. The morphology and structure of the cell may be harmed by an increase in nanoparticles content. The inflammation and necrosis brought on by hepatocyte injury may increase a cell’s permeability. Via the cell membrane, AST and ALT enzymes are released into the body, increasing their concentration in the blood [13]. As a result, the AST and ALT enzymes are signs of liver injury [23]. Xenobiotically induced variations in cellular or biochemical components could occur due to ingested molluscicides [24]. Aspartate and Alanine aminotransferases (AST and ALT) perform a critical role in many stressful conditions [25]. These enzymes AST and ALT were used as biomarker for water pollution in *Helisoma duryi*, *B. alexandrina*, and *Lymnaea natalensis* snails [26].

Therefore, the current study aimed to describe the ecotoxicity of fungal-synthesized SeONPs in aquatic systems as they were naturally synthesized there by different aquatic microorganisms on the freshwater snail *B. alexandrina* and how it affects survival, the concentrations of alanine aminotransferase and aspartate aminotransferase and the structure of the digestive and hermaphrodite glands in order to evaluate the snail’s potential as a bioindicator for SeONPs pollutant. Moreover, in silico molecular docking studies were conducted to analyze the interactions between the ALT and AST and nano-selenium oxide. *D. magna*, which is regarded as a sensitive organism for the detection of aquatic environmental pollution, was used to confirm the nanotoxicity.

## 2. Materials and Methods

### 2.1. Fungal Strain and Selenium Nanoparticles Preparation and Characterization

The strain *Penicillium chrysogenum* MZ945518 was previously isolated from the southern coast of Alexandria, Egypt, and identified using molecular techniques. This fungal strain was used to synthesize SeONPs according to [27]. *P. chrysogenum* was developed in culture flasks containing potato dextrose broth and kept for 7 days at 25 °C. Centrifugation at 10,000 rpm for 10 min separated the mycelia from the culture filtrate. (Sigma, 3-16 PK, Osterode am Harz, Germany). Following that, 15 mL of culture supernatant was mixed with 3 mM sodium selenite (Na_2_SeO_3_), which was then incubated at 40 °C for 30 min until the red color of SeONPs as a soluble form in the liquid was formed. Finally, the myco-fabricated SeONPs were cleaned by spinning them at 10,000 rpm 3 times in double-distilled water for 10 minutes, followed by 48 h of drying at 60 °C.

The myco-synthesized SeONPs were characterized with a Zetasizer analyzer, UV–Visible spectrophotometer, X-ray diffraction, and transmission electron microscope (TEM), according to [27]. A UV-visible spectrophotometer (PerkinElmer Life and Analytical Sciences, CT, Akron, OH, USA) was used to measure the absorption spectra of SeONPs at wavelengths between 400 nm to 800 nm. The average diameter size, distribution, and the zeta potential charges were calculated using Zetasizer Dynamic Light Scattering (DLS) (Zetasizer Nano ZN, Malvern Panalytical Ltd., Malvern, UK), at an angular position of 173° at 25 °C. To assess the crystalline dimensions of the particles, XRD was conducted on a Bruker D8 Discover Diffractometer, USA, with Cu-K radiation (*λ* = 1.54060 Angstrom). The relative intensity data was collected over a 2θ range of 5100°. The chart yielded 2θ values and relative intensities (I/Io), and core minerals were identified using JCPDS carts. Transmission electron images were also taken using a high-resolution transmission electron microscope (HR-TEM; JEOL 2100, Tokyo, Japan) fitted with an electron diffraction pattern. The elemental structure of SeONPs and their functional groups were investigated using Fourier transform infrared spectroscopy (FTIR; PerkinElmer, OH, USA).

### 2.2. Snails

Adult *B. alexandrina* snails (9–11 mm) were kept and adapted at the Theodor Bilharz Research Institute (TBRI) in Giza, Egypt. For snail fecundity, growth, and shell length, snails (10 snails/L) were kept in plastic aquaria (16 × 23 × 9 cm) with 30 mg/L CaCO_3_ (small pieces of chalk) added [28]. In the aquaria, dechlorinated tap water was used with a pH of 7 ± 0.2 and a temperature of 25 ± 2 °C with a 12 h/12 h photoperiod. Blue-green algae (*Nostoc muscorum*), oven-dried lettuce leaves, and Tetramin were fed to the snails. Egg masses were collected using small pieces of 33 foam sheets [29].

### 2.3. Daphnia Magna

*Daphnia magna* has been cultivated in a synthetic freshwater medium in the Hydrobiology Laboratory of the National Research Center’s Water Pollution Research Department.

In 1 L glass beakers containing gravid females (10–20) on a regular basis, the media was changed three times per week. Every day, the offspring were gathered to be employed in the toxicity trials. *Scenedesmus obliquus*, a green microalga, was given to these animals three times per week at a concentration of 14 × 10^7^ coenobia/mL. The photoperiod used to maintain daphnids was 16 h of light and 8 h of darkness at a temperature of 22 ± 2 °C.

### 2.4. Bioassays

#### 2.4.1. Toxicological Impact on *D. magna*

Daphnids aged 24 h to 30 h were divided into groups and subjected to varying doses of SeONPs (0.67, 0.83, 1.00, and 1.33 mg/L, respectively) in 500 mL beakers containing 100 mL of medium in triplicates without the addition of food. The number of living organisms was counted after 24 h and 48 h. A control group was also run at the same time. In this group, 10 daphnids were 24 h old, and were kept in a 500 mL beaker with 10 mL of free SeONPs medium. Using a dissecting microscope, daphnids were examined, and those with no movement of any internal organs were considered dead [30]. The medial lethal concentrations of *D. magna* were determined using probit analysis.

#### 2.4.2. Toxicological Impact on *B. alexandrina* Snails

Sixty adult *B. alexandrina* snails (measuring 9–11 mm in length) were used in the study. Thirty snails in triplicate (10 snails/L) were subjected to 44.15 mg/L of SeONPs for 24 h, then recovered for two weeks [27]. Thirty non-exposed control snails were tested alongside the exposed snails.

##### Snails’ Egg-Laying Capacity Analysis

Snail survival (Lx as a ratio of the correct one at the time of exposure in weeks (x)), egg-laying capacity (Fecundity) (Mx: mean number of eggs/snail/week), and reproductive rates R_0_ (the sum of LxMx during the experimental period) of *B*. *alexandrina* snails were all recorded weekly alongside the control group [31].

##### Tissue Preparation and Biochemical Analysis

The soft tissues of control and treated snails were crushed with two slides before being removed and cleaned from the hard shell parts with forceps. After the soft tissues were formed, they were weighed, and 1 g of each group’s dry tissue was homogenized in a glass Dounce homogenizer with 10 mL of phosphate buffer.

The tissue homogenates were centrifuged for 10 min at 3000 rpm, and the supernatants were utilized for the biochemical assay. The concentrations of alanine aminotransferase (ALT) and aspartate aminotransferase (AST) were assessed using the [32] method. The optical density of the mixture was recorded with water serving as the reference blank at 505 nm. A standard curve was used to calculate the Units per Liter. Total protein was measured according to the method described in [33]. To calculate the total protein concentration, the absorbance of the sample (A_sample_) and standard (A_standard_) against reagent blank at 550 nm. (520–570 nm). The following formula was used, Protein Concentration (g/100 mL) = (A_sample_/A_standard_) × 5. To calculate the albumin concentration, the absorbance of the sample (A_sample_) and standard (A_standard_) against reagent blank at 630 nm. The following formula was used, albumin concentration (g/100 mL) = (A_sample_/A_standard_) × 4. Albumin was analyzed according to [34]. In brief, three tubes were set up, and each received 2.0 mL of the albumin reagent (citrate buffer, pH 4.2, bromcresol green, detergent, and preservative). An amount of 10 µL of the tissue homogenate solution (the sample) were put into the first tube. As a blank, the second tube was used, which only contained albumin reagent. The third tube, containing 2.0 mL of albumin reagent, received 10 µL of albumin as a positive control (standard). The mixture was incubated for 5 min at 37 °C. The absorbance of the sample (A_sample_) and standard (A_standard_) against the reagent blank at 630 nm are used to determine the albumin concentration. Albumin concentration (g/100 mL) = (A_sample_/A_standard_) × 4) was calculated.

##### Histopathological and Immunohistochemical Analysis

Adult *B*. *alexandrina* snails (9–11 mm) were treated with 44.15 mg/L SeONPs for 24 h before recovering for two weeks. According to Carleton et al. [35], dissection and processing of the digestive and hermaphrodite glands were completed. Briefly, the dissected parts were put in 10% formalin for 12 h, then for the dehydration process, different ethanol concentrations (80%, 90%, and 100%, for 3 h each, respectively) were used. After that, they were cleared in xylene and placed in paraffin. Five micrometer sections were cut using a microtome, fixed on slides, dewaxed in xylene, stained with hematoxylin and eosin, and then covered with glass slips using Canada balsam. A Zeiss microscope was used to examine the prepared slides (Carl Zeiss Microscopy GmbH 07,745 Jena, Germany). For immunohistochemical experiments, 4 μm-thick slices of snails were cut from paraffin blocks and placed on positively charged slides (SuperFrost Plus, Menzel-Glaser, Schoemperlenstraße, Germany). On an automated platform (DakoAutostainer Link 48), the slides were stained using anti-mouse proliferating cell antigen (PCNA) and anti-Cyclin D1 antibodies (Santa Cruz Biotechnology, Dallas, TX, USA) at appropriate working dilution of 1:100 [36]. In ten microscopic areas, the ratio of positively stained brown nuclear (PCNA, Cyclin D1) was determined (under Zeiss light microscopy at ×400).

##### Electron Microscope Study

After *B. alexandrina* snails were exposed to 44.15 mg/L SeONPs for 24 h, the digestive gland was removed and cut into small pieces. Then, it was fixed in 2.5% glutaraldehyde in 0.2 M sodium cacodylate solution for three hours at room temperature (pH 7.4), cleaned in the same buffer for four to eight hours at room temperature, and finally post-fixed for two hours at the same temperature in 1% OsO_4_ solution in 0.2 (pH 7.4). Prior to being implanted in an Epon-Araldite solution, tissues were dried in a succession of ethanol concentrations (70%, 80%, 90%, and 95%, and two changes of absolute ethanol, respectively). Ultrathin pieces were made with a diamond knife, stained with uranyl acetate and lead citrate, and examined using an 80 kV JEOL 100CX-II TEM at the Faculty of Agriculture, Cairo University, Egypt.

##### The Molecular Docking Interaction Study

To study the effect of SeO_2_ (SeONPs) in silico, two enzymes secreted from the hepatopancreas of treated snails, ALT and AST, were selected. The structures of these two enzymes were obtained from the Protein Data Bank (PDB) and encoded (ID: 1XI9) for *Pyrococcus furiosus* ALT and (ID: 1AAM) for *Escherichia coli* AST. The Molecular Operating Environment software was used to perform the molecular docking (MOE 2014.09). After determining the best order for the enzymes, hydrogens were added, and partial charges were calculated in order to reduce the energy of the ligand (SeO_2_) molecule.

### 2.5. Statistical Analysis

Lethal concentration levels were established using probit analysis [37]. Using the Student’s *t*-test, the averages of the experimental and control groups were compared [38].

## 3. Results

### 3.1. Selenium Nanoparticles Green Synthesis

**Figure 1:**
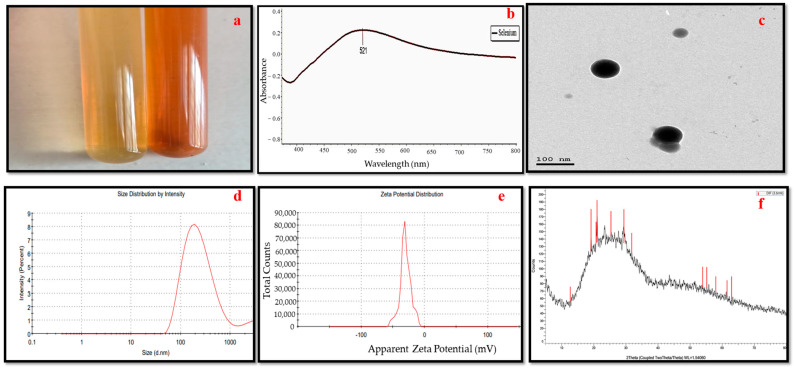
SeONPs Characterization. (**a**) brick-red color of the biogenic selenium oxide nanoparticles. (**b**) UV–Vis spectrum with maximum absorbance at 521 nm. (**c**) TEM photograph of the spherical SeONPs. (**d**) Size distribution. (**e**) Zeta potential. (**f**) XRD pattern of SeONPs.

In the current research, the biomolecules in the culture filtrate of *Penicillium chrysogenum* MZ945518 were shown to be effective at reducing Se ions and producing SeONPs. The transformation of the fungus filtrate’s color from colorless to ruby red after the addition of the precursor solution (Na_2_SeO_3_) served as an indicator for the synthesis of SeONPs (Figure 1a).

### 3.2. Selenium Nanoparticles’ Characteristics

In addition to color change, the fabrication of SeONPs was confirmed by determining the maximum SPR with UV-vis spectroscopy at a wavelength between 200 nm and 800 nm. The maximum absorbance for SeONPs produced by *P*. *chrysogenum* was seen as a single absorbance peak at 521 nm (Figure 1b). Furthermore, the TEM analysis also showed that the developed nanoparticles were round and had crystalline diameters between 44 and 77 nm (Figure 1c). The obtained nano-formulation of the myco-synthesized SeONPs had 204 nm average hydrodynamic diameters and a zeta potential of −30.1 mV (Figure 1d,e). Figure 1f shows X-ray diffraction patterns of the SeONPs, which showed a broad pattern with no Bragg peaks.

### 3.3. Toxicological Impact Assessment

In the current investigation, *D. magna* was used as a bioindicator for SeONPs as it is sensitive to aquatic pollution. According to the recent findings, the half-lethal concentration (LC_50_) of SeONPs for *D. magna* after 24 h was 1.62 mg/L, and after 48 h, it was 0.67 mg/L (Table 1).

**Figure 2:**
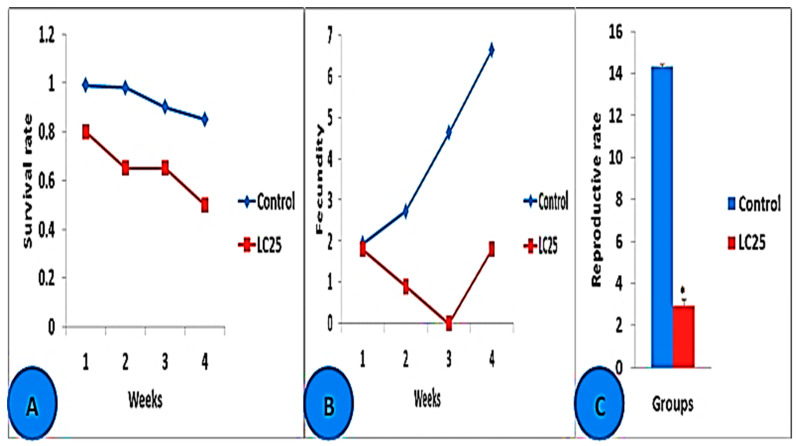
*B. alexandrina* snail survival, fecundity, and reproductive rate after two weeks of exposure to LC25 (44.15 mg/L) SeONPs and two weeks of recovery. (**A**) The rate of survival (Lx). (**B**) The fecundity (Mx). (**C**) *B. alexandrina* snail reproductive rate. * = significant difference from control at *p* < 0.05.

Our previous work showed that a molluscicidal effect against adult *B. alexandrina* snails was observed after being exposed to SeONPs for 96 h at LC_25,_ 44.15 mg/L [27]. According to the results of the current investigation, *B. alexandrina* snails exposed to 44.15 mg/L of SeONPs for 24 h per week for two weeks had considerably lower survival rates (Lx) and fecundities (MX) than those in the control group. Furthermore, exposed snails had a significantly lower reproductive (Ro) rate than the control group (Figure 2A–C).

**Figure 3:**
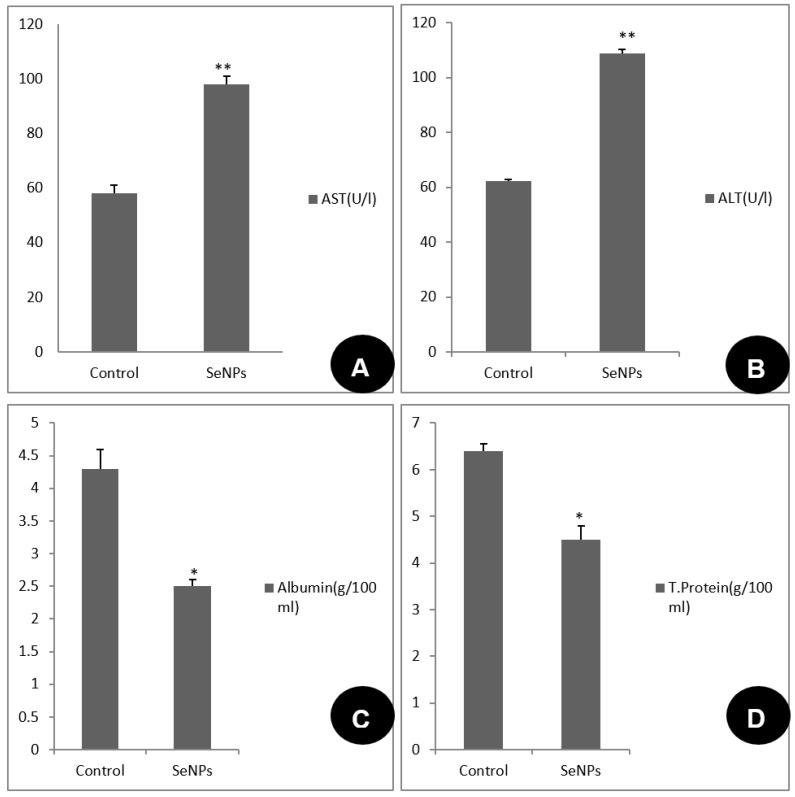
The effect of SeONPs on *B. alexandrina snail* transaminases (AST and ALT)) (**A**,**B**), (**C**) total protein, and (**D**)albumin concentrations. * = significant difference from control at *p* < 0.05. ** = highly significant compared to control at *p* < 0.01.

The SeONPs’ effect on some biochemical studies of *B. alexandrina* was assessed. The current investigation found that the concentrations of total protein and albumin were significantly reduced (*p* < 0.05) in the snails subjected to 44.15 mg/L SeONPs, while the levels of transaminases (AST and ALT) were significantly elevated (*p* < 0.01) in comparison to the control group (Figure 3).

**Figure 4:**
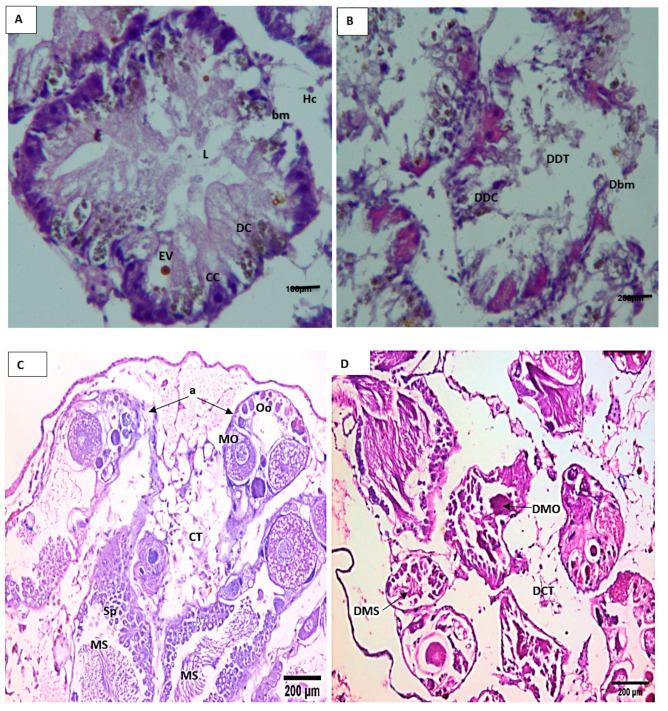
Histopathological sections of *B. alexanderina* snails’ digestive gland and ovotestis region. (**A**) The digestive gland lumen (L) of an untreated *B. alexanderina* digestive gland was surrounded by the basement membrane (bm) and three different cells: calcium cells (CC), digestive cells (DC), and excretory vesicle (Ev). (**B**) Exposed *B. alexanderina* snails to 44.15 mg/L of SeONPs, showing degenerated digestive tubules (DDT), basement membrane (Dbm), and digestive cells (DDC). (**C**) Section from an untreated snail showing the hermaphrodite gland with many acini (a) containing mature sperm (MS) at the center of acini and spermatocytes (Sp), while female acini showing mature ovum (MO) and Oocytes (Oo) and well-formed connective tissue between acini (CT). (**D**) Section from exposed snail to 44.15 mg/L of SeONPs showing the hermaphrodite gland with deformed connective tissue (DCT), and destructed mature ovum (DMO) and sperms (DMS).

**Figure 5:**
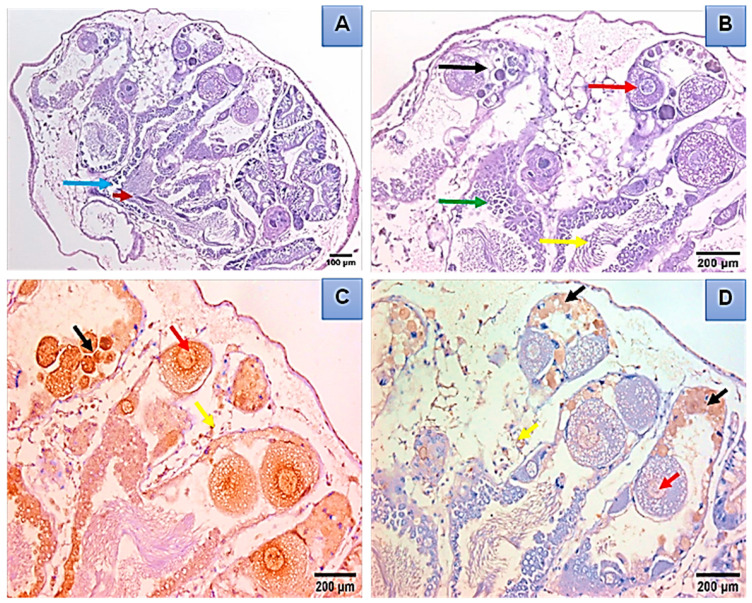
The hermaphrodite gland of *B. alexanderina* snails by light microscope. (**A**,**B**) mature ovum (red arrow), oocytes (black arrow), sperms (yellow arrow), spermatocytes (green arrow), primary oocytes (blue arrow), and connective tissue (dark red arrow). (**C**) Control hermaphrodite gland, demonstrating PCNA expression in the ovum (red arrow) and in oocytes (black arrow), in the interstitial cells (yellow arrow), as brownish positive nuclei. (**D**) Normal control showing cyclin D1 expression in the ovum (red arrow) and in oocytes (black arrow), in the interstitial cells (yellow arrow) as brownish positive nuclei.

The light microscopy showed that the digestive gland of control *B. alexandrina* snails consisted of many follicles, and each follicle was lined by a basement membrane and has three cell types: digestive, excretory, and calcium cells (Figure 4A). The digestive gland tissues were damaged after being exposed to 44.15 mg/L of SeONPs. Such effects included follicle and basement membrane degeneration, and the destruction of digestive, excretory, and calcium cells, and an increase in follicle lumen (Figure 4B). The normal hermaphrodite gland of *B. alexandrina* is primarily made up of female oogenic cells and male reproductive cells (Figure 4C). Gonadal cells of exposed snails to 44.15 mg/L SeONPs experienced severe damage, including connective tissue deformation, mature ova destruction, and sperm degradation (Figure 4D). Immunohistochemistry of the normal hermaphrodite gland showed positive expression of both PCNA and Cyclin D1 in the ovum, oocytes, and interstitial cells as brownish positive nuclei (Figure 5A–D).

**Figure 6:**
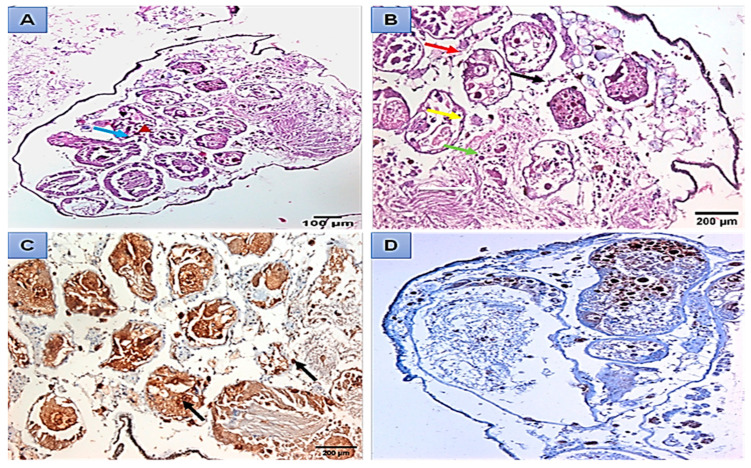
Light micrographs of the hermaphrodite gland of *B. alexanderina* snails exposed to 44.15 mg/L of SeONPs. (**A**,**B**) Degenerated ovum (blue arrow), degenerated oocytes (head arrow), Oocytes (yellow arrow), Sperms (white arrow), Spermatocytes (green arrow), connective tissue (black arrow), space between acini (red arrow). (**C**) Section in the hermaphrodite gland demonstrating positive expression of PCNA (70%) in the ova, interstitial cells, and oocytes (black arrow). (**D**) Section in the hermaphrodite gland revealing positive expression of cyclin D1 (30%) in interstitial cells, ova, and oocytes (black arrow).

There were negative changes in the snails’ hermaphrodite glands after 44.15 mg/L SeONPs exposure (Figure 6A,B). PCNA immunohistochemistry revealed that at least 70% of the nuclei in sperm and ova were brownish in color (Figure 6C). Cyclin D1 was also found to be expressed in sperm, ova, and interstitial cells (Figure 6D).

**Figure 7:**
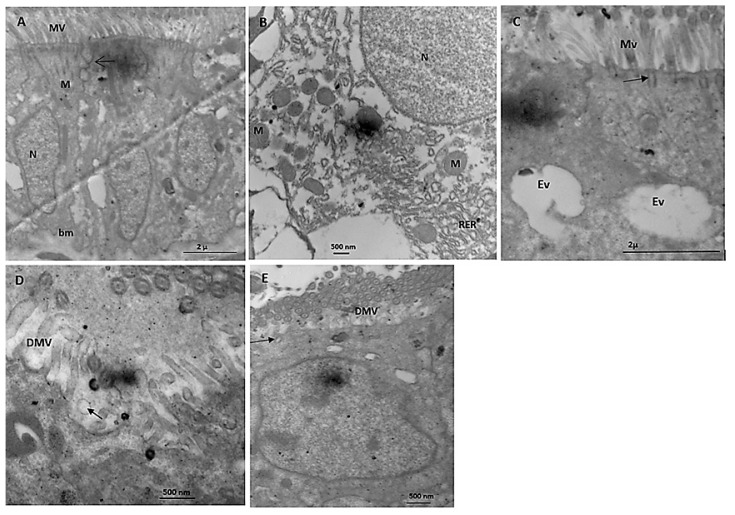
Electron micrographs of *B. alexandrina* snails’ digestive glands. (**A**) Control adjacent digestive cells showing an apical region with microvilli (MV), mitochondria (M), and nuclei (N) near the basement membrane (bm). Note that a tight junction binds two neighboring cells (arrows). (**B**) Part of the digestive cell showing the nucleus (N), numerous mitochondria (M), and rough endoplasmic reticulum (RER). (**C**) Excretory cells of untreated *B. alexandrina* snails showing excretory vacuole (Ev) and microvilli (MV) at the apical region. Arrow refers to the rootlet of a microvillus. (**D**) The apical region of treated digestive cells with 44.15 mg/L of SeONPs showing destroyed microvilli (DMV) and presence of surface bleb (arrow). (**E**) Treated digestive cell with LC_25_ SeONPs showing destroyed microvilli (DMV) and destroyed rootlet of a microvillus (arrow).

Based on an electron microscopy study, the digestive gland of *B. alexandrina* control snails has numerous microvilli at the apex of the digestive cells, and normal nuclei located near the basement membrane (Figure 7A). Tight junction at the upper lateral zone bound adjacent digestive cells. Many mitochondria were distributed near the nucleus of the digestive cell and the rough endoplasmic reticulum (Figure 7B). The excretory cells of the digestive glands showed two excretory vacuoles and many rootlets of the microvilli (Figure 7C). The digestive cells of *B. alexandrina* snails showed partial destruction of the apical microvilli and the presence of some surface blebs after being exposed to 44.15 mg/L of SeONPs (Figure 7D). Partially destroyed microvilli rootlets appeared under the destroyed microvilli (Figure 7E).

### 3.4. Docking Study

**Figure 8:**
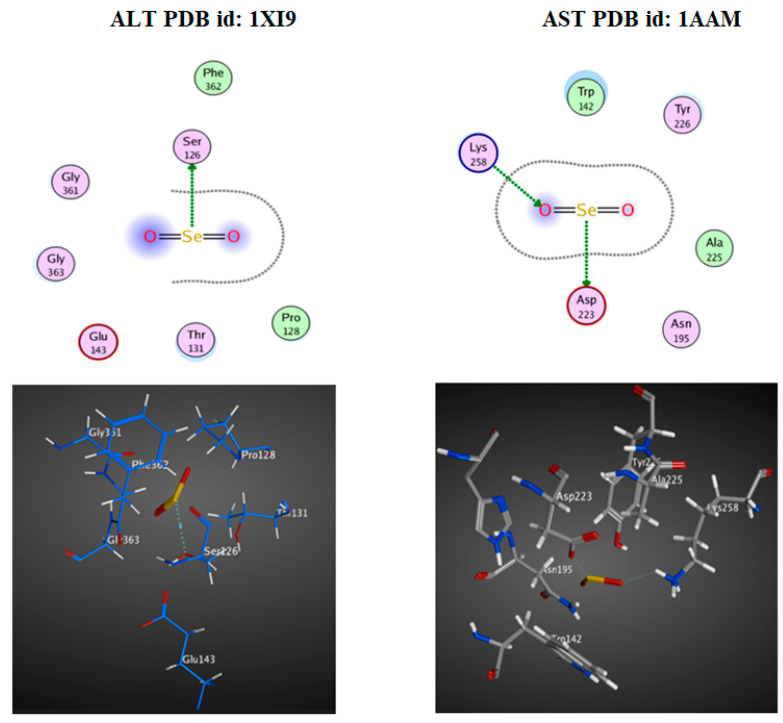
3D and 2D docked interaction map for the SeO_2_ (SeONPs) with the binding sites of ALT and AST.

ALT and AST, the two hepatopancreatic enzymes, were chosen to investigate the action of selenium oxide (SeONPs). The docking interaction demonstrated a high efficiency for SeO_2_ against the ALT and AST receptor binding sites (Figure 8). The docking score, also called interaction-free energy, was used to examine the enzymes’ effect. Selenium oxide was able to dock with these enzymes through their H-interaction scores (−1.0, −3.8, and −2.3 Kcal/mol) against ALT and AST, respectively (Table 2).

## 4. Discussion

Selenium is a vital mineral for health that controls a number of cellular functions through selenium proteins. A variety of disorders, including infectious diseases, can be prevented with selenium. Despite all these advantages, selenium in high dosages might have undesirable side effects. Reports now concentrate on employing nanoparticles to avoid excessive dosages of selenium while maintaining biological benefits [39]. Nano-selenium (SeNPs) has many advantages, such as its small size and higher activity, in addition to the ability to use selenium in its zero oxidation state (Se^0^) [40]. The aquatic invertebrates, such as gastropods, bivalves, and crustaceans are good model organisms for studying the nanomaterials toxicological effects [41]. Additionally, *Daphnia magna* is extremely susceptible to contaminants and serves as a model organism for bioindicators in ecotoxicology [18,42]. This model may help detect the acute toxicity of tested materials with *Daphnia* spp., because it is simple, quick, and has a distinct ending [43].

The present results indicated that SeONPs have a toxicological effect on *D. magna*. SeONPs had a half-lethal concentration (LC_50_) of 1.62 mg/L after 24 h and 1.08 mg/L after 48 h on *D. magna*. These findings were in a good accordance with Dunbar et al. [44], who studied the toxic impacts of sodium selenite on *Daphnia magna Straus* and stated that the acceptable maximum toxicant concentration of 1.73 mg/L (<2.0 mg/L Se). Additionally, In their study of the toxicity of 50 metals on *Daphnia magna*, Okamoto et al. [45] found that 13 elements (Al, Sc, Cr, Co, Ni, Zn, Se, Rb, Y, Rh, Pt, Tl, and Pb) had half lethal values between 100 µg L^−1^ and 1000 µg L^−1^. Boyum [46] reported similar findings and indicated that the acute 48 h LC_50_ of sodium selenate for *Daphnia pulicaria* and *Daphnia magna* was 1.01mg Se/1 and 0.25 mg Se/1, respectively. For *D. magna* and *D. pulicaria*, the sodium selenite 48 h LC/sub-50/values were 0.45 mg Se/1and 0.006 mg Se/1, respectively. Daphnids collected Se quantities in the water that might be poisonous and negatively impact fish or seabirds along the food chain [47]. Similarly, ref. [48] found that SeNPs were toxic to the microalgae *Chaetoceros gracilis* in a time and concentration dependent manner. Moreover, ref. [49] observed that the use of nano-selenium improved the growth performance of the Nile *tilapia* in 1 mg/kg concentration group while this growth performance was low in 2 mg/kg Se-Nps concentration, thus they concluded that SeONps supplementation may lead to toxic effects in over-dose concentrations. Additionally, Ibrahim et al. [50] concluded that the nanomaterials could negatively affect the membrane permeability and structure, leading to the death of the aquatic creatures.

Morad et al. [27] stated the molluscicidal activity of the myco-synthesized SeNPs against *Biomphalaria alexandrina* snails with sublethal concentration (LC_25_) 44.15 mg/L. The current investigation revealed that exposure of *B. alexandrina* snails to 44.15 mg/L of SeONPs decreased the survival rate, fecundity, and reproductive (R_o_) rate compared to the control group. These parameters were the most significant toxicological endpoint tests for evaluating the nanomaterials’ toxicity [50]. Nano-metals caused deleterious effects in the reproductive system, kidney, liver, brain, and other body systems [51] through inducing of releasing of reactive oxygen that caused damages of cells decreased fertility, delayed in organism development, and subsequently the death of this snail [52,53]. Ibrahim and Sayed [54] correlated these reductions with the severe damages in the digestive and hermaphrodite gland cells of treated snails. Oliveira-Filho et al. [55] stated that silver nanoparticles caused deleterious effects on reproduction and survival rates of *B. glabrata* snails.

The present results showed that the activities of the transaminases (AST and ALT) in snails exposed to 44.15 mg/L of SeONPs were significantly increased, while there was a reduction in total protein and albumin concentrations compared with the control group. The increase in ALT and AST after exposure was due to the great damages of SeNPs on the hepatic cells [25,56]. The decrease in protein might be due to the animals’ effort to restore normal levels after the destructive effect of SeONPs on protein biosynthesis [57]. The major cause of the decline in albumin level after exposure may be due to the deleterious effect of SeNPs on liver parenchyma [52].

The digestive gland is composed of “digestive” cells that are responsible for the absorption and digestion of food and “secretory” cells that produce digestive enzymes and calcareous concretions. In between these two cell types, there are scattered undifferentiated cells [58]. The present results showed histopathological damage in the digestive and hermaphrodite glands, where, after the exposure to SeONPs, there was degeneration of the digestive cells and excretory cells. Additionally, there was an increase in the lumen of tubules. Regarding the hermaphrodite gland of *B. alexandrina,* it was comprised of the male reproductive cells and the female oogenic cells. Exposing snails to 44.15 mg/L of SeONPs caused great damage to gonadal cells; the connective tissue was deformed, the mature ova were destroyed, and the sperm were degenerated. These histopathological alterations might be related to the direct toxic effects of the heavy metals on the different organs of the target animals [59]. Similarly, Abdel-Tawab et al. [16] reported severe damages in the digestive and hermaphrodite glands of *B. alexandrina* snails after exposure of to 314.5 mg/L of Cerium oxide nano-composite; furthermore, there were degeneration and rupture of sperms and ova. Additionally, in the digestive gland, there were ruptures, degeneration, and vacuolation of some digestive cells, in addition to a marked increase in the number of the secretory cells.

Specific antibody-antigen interactions were used in immunohistochemistry to demonstrate antigens in tissue sections [60,61,62]. Cyclin D1 is a cell cycle progression regulator. Its upregulation may be associated with cancer [63,64,65]. PCNA could be used as an important marker for hepatotoxicity, with more PCNA-positive cells indicating severe and necrotic cell damage [66], with more PCNA-positive cells indicating severe and necrotic cell damage [67].

Immunohistochemistry of a normal hermaphrodite gland showed that both PCNA and Cyclin D1 were expressed as brownish nuclei with a low percentage. PCNA expression was ±70% positive in sperm and ova after 44.15 mg/L SeNPs exposure. Cyclin D1 was also found to be expressed (±30%) in sperm, ova, and interstitial cells. Similarly, Ibrahim et al. [36] revealed that after being exposed to LC_25_ of *Nerium oleander* methanolic extract, there was degeneration and destruction in some ova and sperm, and immunohistochemistry for PCNA expression was positive in ±30% of sperm and ova.

Numerous cilia were visible in electron photomicrographs of the digestive glands of *B. alexandrina* snails, as were microvilli at the apex of the digestive cells, normal nuclei based near the basement membrane, rough endoplasmic reticulum, and many mitochondria. The excretory cells showed two excretory vacuoles and many rootlets of microvilli. The digestive cells of *B. alexandrina* snails showed partial destruction of the apical microvilli and the presence of some surface blebs after being exposed to 44.15 mg/L of SeONPs. These results are in accordance with Yousef and EI-Kassas [68], who reported cytoplasmic fragmentation, vacuolation, and toxic agent accumulation in the cytoplasm of *B. alexandrina* digestive and excretory cells after exposure to *E. splendens*-plant. In addition, Hamed et al. [69] discovered ultrastructural changes in the digestive gland of *Eobania vermiculata* snails after topical application of two carbamate molluscicides. Disruption and reduction in microvilli, and the formation of surface blabs, cytoplasmic vacuolization, and increases in the number of calcium and excretory cells, were among the changes. Receptor-ligand interactions using molecular docking is an interesting method for predicting the role of ligand compounds in the inhibition or activation actions of some enzymes. The enzymatic mechanisms included ALT (PDB id: 1XI9 [70]) and AST (PDB id: 1AAM [70]. ALT and AST are secreted in the body to metabolize protein and produce energy; in addition, they are important indicators of liver disorders [71]. The current findings showed potential SeO_2_ effect that reacted with ALT and AST and caused interfering with their active amino acid catalytic sites.

## 5. Conclusions

The present study investigated the toxicity of mycosynthesized selenium oxide nanoparticles (SeONPs) in many aspects regarding the intermediate host *Biomphlaria alexandrina* of *Schistosoma mansoni* parasite. The *B. alexandrina* snails’ rates of survival, fecundity, and reproduction may be positively reduced by SeONPs. The biochemical analysis revealed a significant increase in the digestive gland enzymes, such as AST and ALT, while there was a noticeable reduction in the concentration of albumin and total protein. The use of in silico molecular docking revealed a potential SeONPs impact that interacted with the active amino acid catalytic sites of ALT and AST. The histological and immunohistochemical results showed great defects in the structure of digestive and ovotestis glands after the exposure to SeONPs. In addition, the transmission electron microscope examination revealed abnormalities in the digestive gland cells’ fine structure. As an indicator model of freshwater pollution, *D. magna* showed high sensitivity to low concentrations of SeONPs. The results of the present investigation represent, to our knowledge, the first ecotoxicological evaluations of SeONPs on the freshwater snail *B. alexandrina*. In conclusion, this snail can be used as a biomonitor for SeONPs as a freshwater pollutant. To determine if it is currently necessary to regulate and/or manage the use and discharge of SeONPs in the Nile River, more research is required to assess the current contamination levels of SeONPs in Egyptian aquatic ecosystems.

## Figures and Tables

**Table 1:** Toxicity of SeONPs on *Daphnia magna* after 24 h and 48 h of exposure.

Tested Animals	LC_10_ (mg/L)	LC_25_ (mg/L)	LC_50_ (mg/L)	LC_90_ (mg/L)
***D. magna*** **/24 h**	0.70	1.04	1.62	3.77
***D. magna*** **/48 h**	0.42	0.53	0.67	1.08

**Table 2:** In silico docking study of hepatopancreas enzymes, ALT, and AST with the ligand selenium oxide.

PDB ID	Docking Score (Kcal/mol)	Interaction Type	Amino Acid Residue Involved in Docking
**ALT (1XI9)**	−1.0	H-donor	SER 126
**AST (1AAM)**	−3.8	H-donor	ASP 223
	−2.3	H-acceptor	LYS 258

## Data Availability

All data generated or analyzed during this study are included in this article.
